# Ultrastructure of spermatozoa in three cicada species from China (Hemiptera, Cicadomorpha, Cicadidae)

**DOI:** 10.3897/zookeys.776.26966

**Published:** 2018-07-26

**Authors:** Beibei Cui, Cong Wei

**Affiliations:** 1 Key Laboratory of Plant Protection Resources and Pest Management, Ministry of Education,; 2 College of Plant Protection, Northwest A&F University, Yangling, Shaanxi 712100, China

**Keywords:** Cicadoidea, Cicadomorpha, Hemiptera, Insecta, morphology, sperm

## Abstract

The ultrastructure of mature spermatozoa of three cicada species, *Subpsaltria yangi*, *Karenia caelatata*, and *Platypleura kaempferi*, was investigated using epifluorescence and transmission electron microscopies. This is the first investigation of the sperm ultrastructure of species in the subfamily Tibicininae and the tribe Sinosenini, represented by *S. yangi* and *K. caelatata*, respectively. The three species all produce two or three types of spermatozoa with various lengths, viz., polymegaly. The centriolar adjunct of spermatozoa in *S. yangi* shows a granular substructure, which is different from that of other cicada species, suggesting that spermatozoa in Tibicininae may have their own characteristics in comparison with other cicadas. The centriolar adjunct of spermatozoa of *K. caelatata* displays characteristics similar to that of the Cicadinae. Combined with other morphological characters, it is reasonable to remove *K. caelatata* and its allies (i.e., Sinosenini) from Cicadettinae to Cicadinae. The study of sperm ultrastructure, particularly in the species of Tibicininae and Sinosenini, expands the spermatological research of Cicadidae and provides more information for phylogenetic analysis of Cicadidae.

## Introduction

As germ cells, sperm are evolving at the fastest speed and are among the most diverse cell types with the highest degree of variation in insect growth (Baccetti and Afzelius 1976, Jamieson 1987, Joly et al. 1989). Spermatological characteristics have been used for distinguishing taxa and for clarifying phylogenetic relationships of related taxa (Jamieson 1987, 1991, Jamieson et al. 1999, Lino-Neto and Dolder 2001, Zhang and Hua 2017). The sperm structure of insects, similar to those of other metazoans, is broadly the same, but each group has unique characteristics. Most of these generally tiny, motile spermatozoa are slender with a single flagellum, but some have more than one flagellum (Chawanji et al. 2005, 2006). The flagellum is the power source for sperm mobility (Ciolfi et al. 2016).

There are two aspects of insect sperm, the length and structure, together revealing any sperm polymorphism. The sperm length in Diptera extends across a great range (Joly et al. 1991, Joly et al. 1995). It has been reported that two types of nuclei of distinctly discrete lengths are produced in some species of Drosophilidae and Diopsidae (Diptera) (Snook et al. 1994, Pasini et al. 1996, Presgraves et al. 1999). Another aspect is nucleation. Males in butterflies and moths (Lepidoptera) can produce nucleated eupyrene sperm and non-nucleated apyrene sperm; the former has the ability to fertilize eggs, while the exact function of the latter is still uncertain (Katsuno 1977, Silberglied et al. 1984, Osanai et al. 1989, Friedländer 1997, Yamashiki and Kawamura 1997, Kubo-Irie et al. 1998, Watanabe and Bon’no 2001, Friedländer et al. 2005, Hayakawa 2007). There exists an extreme case in *Dahlbominus fuscipennis* (Hymenoptera, Eulophidae) which has at least five different types of spermatozoa with diverse appearances (Lee and Wilkes 1965). Although the function of polymorphic sperm in insects remains uncertain, some authors suggest that it may be related to sperm competition (Snook 1998, Swallow and Wilkinson 2002).

The family Cicadidae of the order Hemiptera includes approximately 3,000 extant species worldwide, and about 210 extant species distributed in China (Sanborn 2013, Chou et al. 1997). This family includes four subfamilies, i.e., Cicadinae, Cicadettinae, Tibicininae and Tettigomyiinae (Marshall et al. 2018). Up to the present, studies on the sperm ultrastructure of Cicadidae have addressed 13 species (Snook et al. 1994, Pasini et al. 1996, Presgraves et al. 1999, Kubo-Irie et al. 2003, Chawanji et al. 2005, 2006, Chawanji et al. 2007). It was found that *Graptopsaltria nigrofuscata* (Cicadinae) can produce two types of spermatozoa, but only the longer spermatozoa have fertility (Kubo-Irie et al. 2003). Chawanji et al. (2005) revealed polymegaly in spermatozoa of all four investigated species of African platypleurine cicadas of the subfamily Cicadinae. The sperm ultrastructure of five cicadas, currently belonging to Cicadettinae and Tettigomyiinae, respectively, has also been studied (Chawanji et al. 2006). Such scant information is applicable to phylogenetic study of Cicadidae from the point of view of spermatology. However, studies on sperm structure in Cicadidae are still insufficient, particularly for some taxa whose systematic status remains controversial, and for the subfamily Tibicininae, of which the sperm structure has never been investigated in any species.

Herein, the sperm ultrastructure of three cicada species were observed using both epifluorescence and transmission electron microscopies (TEM). The sperm ultrastructure of *Subpsaltria yangi* is the first detailed description of spermatozoa investigated in the subfamily Tibicininae. We also address the systematic placement of the genus *Karenia* based on a comparison of the sperm ultrastructure of this species and other species. In addition, coupled with previous studies, we discuss the similarities and differences in sperm ultrastructure among different subfamilies of Cicadidae, aiming to provide useful clues for taxonomic and phylogenetic studies of the Cicadoidea.

## Materials and methods

Male adult cicadas were collected using a net. Their identities and detailed collecting information are shown in Table 1. The higher classification of Cicadidae follows that of Marshall et al. (2018).

**Table 1.** Taxonomic status and collecting information of three investigated species.

**Species**	**Subfamily**	**Collecting sites**	**Collecting dates**
*Subpsaltria yangi* Chen	Tibicininae	Helan Mountains, Ningxia, China	11–16 June 2016
*Karenia caelatata* Distant	Cicadinae	Ankang, Shaanxi, China	9–15 August 2016
*Platypleura kaempferi* (Fabricius)	Cicadinae	Yangling, Shaanxi, China	23 June–12 July 2016

### Sample preparation and epifluorescence microscopy observation

Samples (at least five individuals of each species) were anesthetized with alcohol at a concentration of 75%, and dissected with a fine scalpel blade under a binocular microscope (Olympus SZX16, Olympus Corporation, Tokyo, Japan) to obtain the seminal vesicles from which spermatozoa were recovered. For measuring the sperm total length, sperm samples were placed in 1% bisbenzimidazole Hoechst 33258, a cell-permeable adenine–cytosine binding epifluorescent dye used to stain DNA (Sakaluk and O’Day 1984), for 1 min, then rinsed in three changes of 0.1 M phosphate buffered saline (PBS, pH 7.2). Spermatozoa were evenly spread on a microscope slide and covered with a coverslip. Slides were examined with an Olympus BX-51 epifluorescence microscope (Olympus Corporation, Tokyo, Japan) at a wavelength of 343 nm. Digital images of 50–100 spermatozoa were randomly captured from each species under the same microscope with an Olympus DP72 camera (Olympus Corporation, Tokyo, Japan). Sperm lengths were measured using the Olympus DP2-BSW software version 2.1.

### Sample preparation and transmission electron microscopy observation

Sperm samples were fixed in 2.5% glutaraldehyde (0.1 M PBS, pH 7.2) for 12 h at 4 °C, and the materials were rinsed with 0.1 M phosphate buffered saline (PBS, pH 7.2), then fixed in 1% osmium tetroxide for 2 h at room temperature. Alcohol dehydration with a concentration gradient was performed after rinsing with the same PBS. Treated samples were then embedded in Epon 812 resin. A diamond knife was used to obtain ultrathin sections which were collected on 300 mesh copper grids before staining with uranyl acetate and lead citrate. Sections were examined and photographed with a HT7700 transmission electron microscope (HITACHI, Tokyo, Japan) at 80 kV.

### Data analysis

The measured data were recorded using Microsoft Excel version 2010. Then an analysis of variance was conducted to verify the mathematically significant differences in sperm length between different sperm types within species using SPASS version 19. Correlation analysis between nucleus length and tail length was performed using R version 3.3.2. Measurements are reported as mean ± standard error.

## Results

### Epifluorescence Morphology

**Figure 1.**
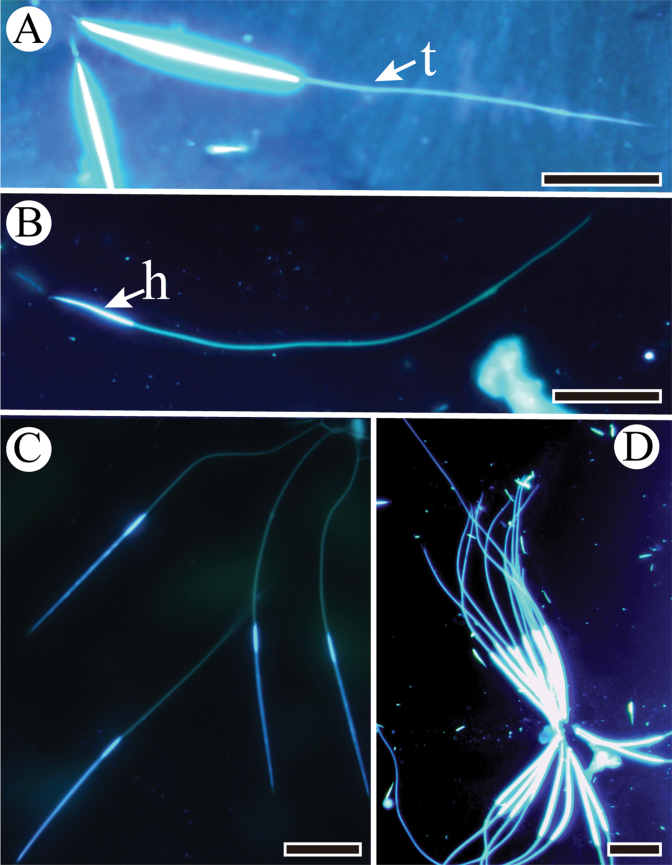
Epifluorescent microscope images of spermatozoa stained with Hoechst 33258. **A** Spermatozoon of *Subpsaltria yangi* with a head and a tail (t) **B** Spermatozoon of *Platypleura kaempferi* with a short head (h) and an elongated tail **C** Slender spermatozoa of *Karenia caelatata* with a head and a tail **D** Spermatozoa of *S. yangi* aggregated into bundles. Scale bars: 20 μm.

Mature spermatozoa of the three species are all linear with needle-like heads and long tails that tapered posteriorly (Figure 1A–C). The heads of the spermatozoa are aggregated into bundles, with the thread-like tails scattered freely (Figure 1D).

Sperm morphologies of the three species are similar, but the spermatozoa vary in length. Based on their remarkably different total length, spermatozoa within a species are divided into disparate types (Table 2). In *K. caelatata*, the spermatozoa can be divided into three types: long spermatozoa, medium spermatozoa, and short spermatozoa. The spermatozoa of the other two species, *P. kaempferi* and *S. yangi*, can be classified into two types: long spermatozoa and short spermatozoa. Within each species, the total lengths of different types of spermatozoa are significantly different (P < 0.01; one-way ANOVA). Among the long spermatozoa of these three species, the spermatozoa of *K. caelatata* is the longest (178.45 ± 10.82 μm); among the short spermatozoa, the spermatozoa of *S. yangi* is the shortest (64.36 ± 5.13 μm) (Table 2).

**Table 2.** Total sperm length (μm) (mean ± SE) of three cicada species.

**Species**	**Length range**	**Length of long spermatozoa**	**Length of medium spermatozoa**	**Length of short spermatozoa**	**N**
*Subpsaltria yangi*	55.82–110.58	105.90 ± 2.96	–	64.36 ± 5.13	74
*Karenia caelatata*	83.25–195.34	178.45 ± 10.82	117.13 ± 4.43	88.83 ± 2.15	99
*Platypleura kaempferi*	68.91–125.21	111.51 ± 9.46	–	89.35 ± 5.76	49

Additionally, the differences in total length of spermatozoa and the sizes of sperm nuclei and tails both within and between species are also significantly different. In *S. yangi*, the lengths of nuclei fall into two classes, and the lengths of tails present three classes. There is a weak correlation between the nucleus and tail lengths in *S. yangi* (Table 3). In *K. caelatata*, the lengths of nuclei fall into two classes; the lengths of tails fall into two clear classes. There is a moderately correlation between the nucleus and tail lengths in *K. caelatata* (Table 3). Sperm nuclei of *P. kaempferi* can be classified into two classes according to their length; the tail lengths fall into three classes. The nucleus length is moderately correlated to the tail length in this species (Table 3).

**Table 3.** Modal classes and correlation coefficients (r) of nucleus length (mean ± SE μm) vs tail length (mean ± SE μm) in the spermatozoa of three cicada species.

**Species**	**Length of short nucleus**	**Length of long nucleus**	**Length of short tail**	**Length of median tail**	**Length of long tail**	**r**	**N**
*Subpsaltria yangi*	16.79 ± 5.40	42.09 ± 4.03	31.57 ± 3.48	61.31 ± 8.56	102.67± 17.00	-0.24	74
*Karenia caelatata*	19.49 ± 4.75	47.19 ± 3.28	64.00 ± 5.30	–	122.88 ± 11.60	-0.53	99
*Platypleura kaempferi*	18.46 ± 2.80	32.85 ± 3.10	37.67 ± 4.83	56.75 ± 5.80	100.31 ± 8.28	-0.40	49

### Ultrastructure

*Subpsaltria yangi* Chen, 1943

**Figure 2.**
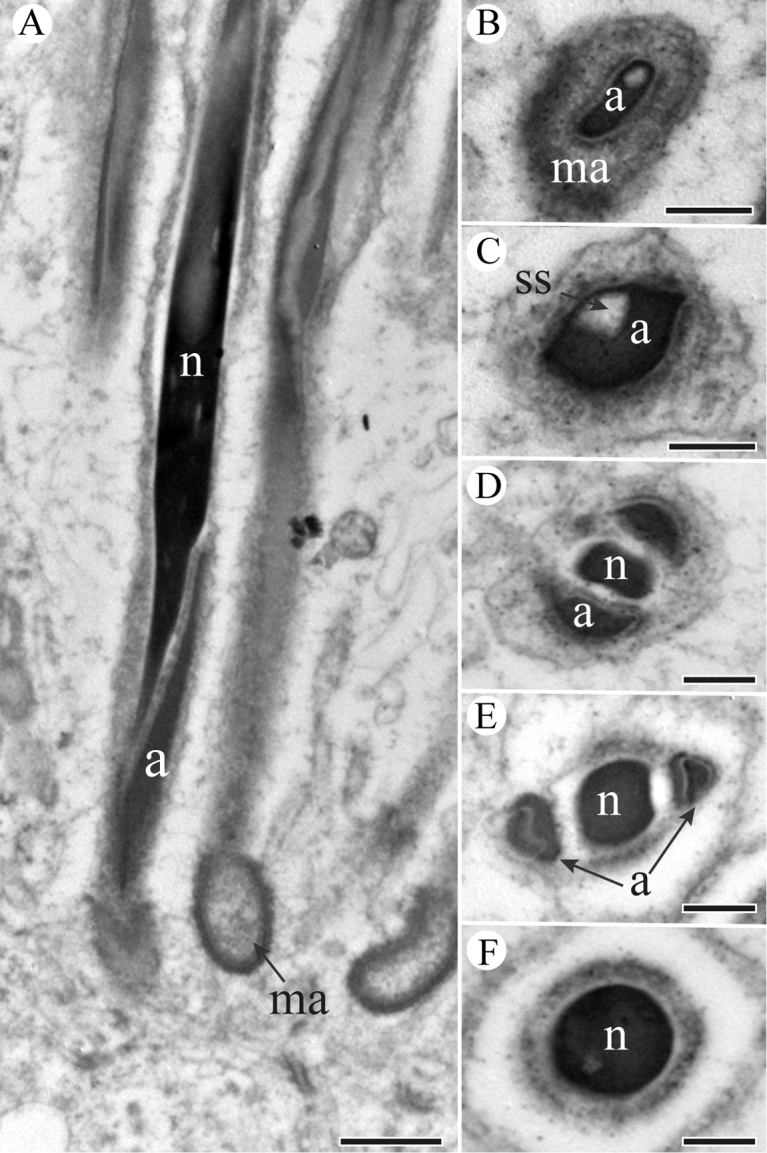
TEM micrographs of *S. yangi* sperm head region. **A** Longitudinal section of sperm head, showing the head region (including acrosome (a) and nucleus (n)) inserted into a homogenous matrix (ma) **B** Cross-section through the tip of acrosome (a), showing acrosome is surrounded by a homogenous matrix (ma) **C** Cross-section through the mid-acrosome (a), showing acrosome (a) and subacrosomal space (ss) **D** and **E** Cross-sections of base of acrosome (a), showing nucleus (n) and two acrosomal processes **F** Cross-section through circular nucleus (n). Scale bars: 500 nm (**A**), 200 nm (**B–F**).

**Figure 3.**
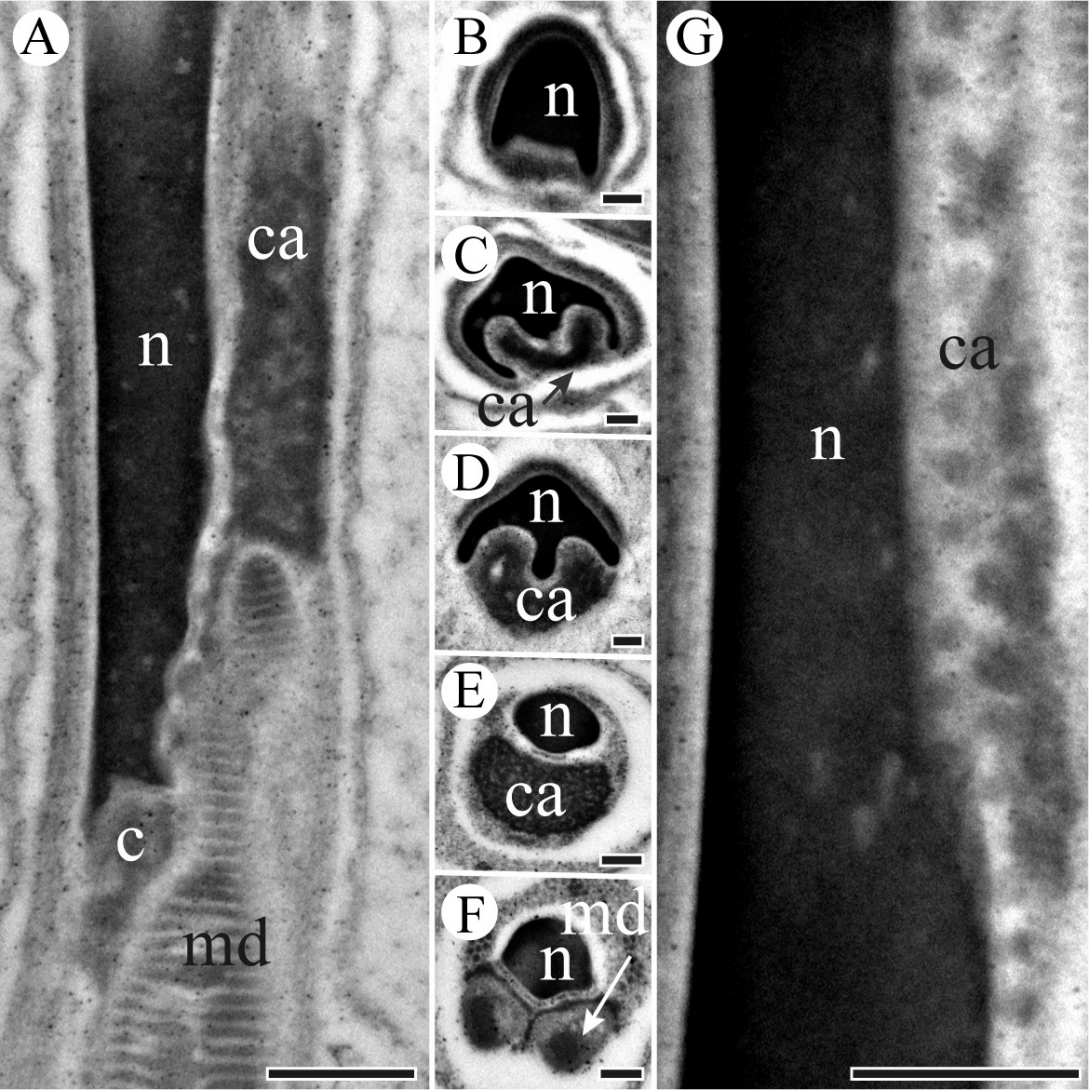
TEM micrographs of *S. yangi* sperm neck region. **A** Longitudinal section of the neck region, showing nucleus (n), centriole (c), granular centriolar adjunct (ca) and mitochondrial derivatives (md) **B** Cross-section anterior of the neck region, showing nucleus (n) **C** and **D** Cross-sections of the mid-neck region, showing nucleus (n) and centriolar adjunct (ca) **E** Cross-section through the terminal end of neck region, showing an eliptical nucleus (n) and a granular centriolar adjunct (ca) **F** Cross-section through the terminal end of neck region, showing a nucleus (n) and two mitochondrial derivatives (md) **G** Magnified longitudinal section of neck region, showing granular centriolar adjunct (ca) next to nucleus (n). Scale bars: 500 nm (**A, F, G**), 200 nm (**B–E**).

The head region that is embedded into a homogenous matrix consists of an acrosome and a nucleus, and the anterior section of nucleus intrudes into an invagination of the acrosome as shown in longitudinal section (Figure 2A). The acrosomal contents are differentiated internally with tubular substructures (Figure 2B–F). The acrosome has two processes with an extension on both sides of the anterior section of the nucleus, and gradually widens in diameter in cross-sections (Figure 2D and E). The nucleus appears to have a cylindrical profile (Figure 2A). The nucleus accommodates to the processes of the acrosome, and is bilaterally concave (Figure 2D and E), finally becoming circular shape in a cross-section (Figure 2F). There is a shallow invagination in the post-lateral part of the nucleus, where the granular centriolar adjunct is located in longitudinal sections (Figure 3A, G). The centriolar adjunct is limited by the invagination of the nucleus, and its shape changes in different cross sections (Figure 3B–E). The diameter of nucleus decreases towards the base of nucleus (Figure 3B–F).

**Figure 4.**
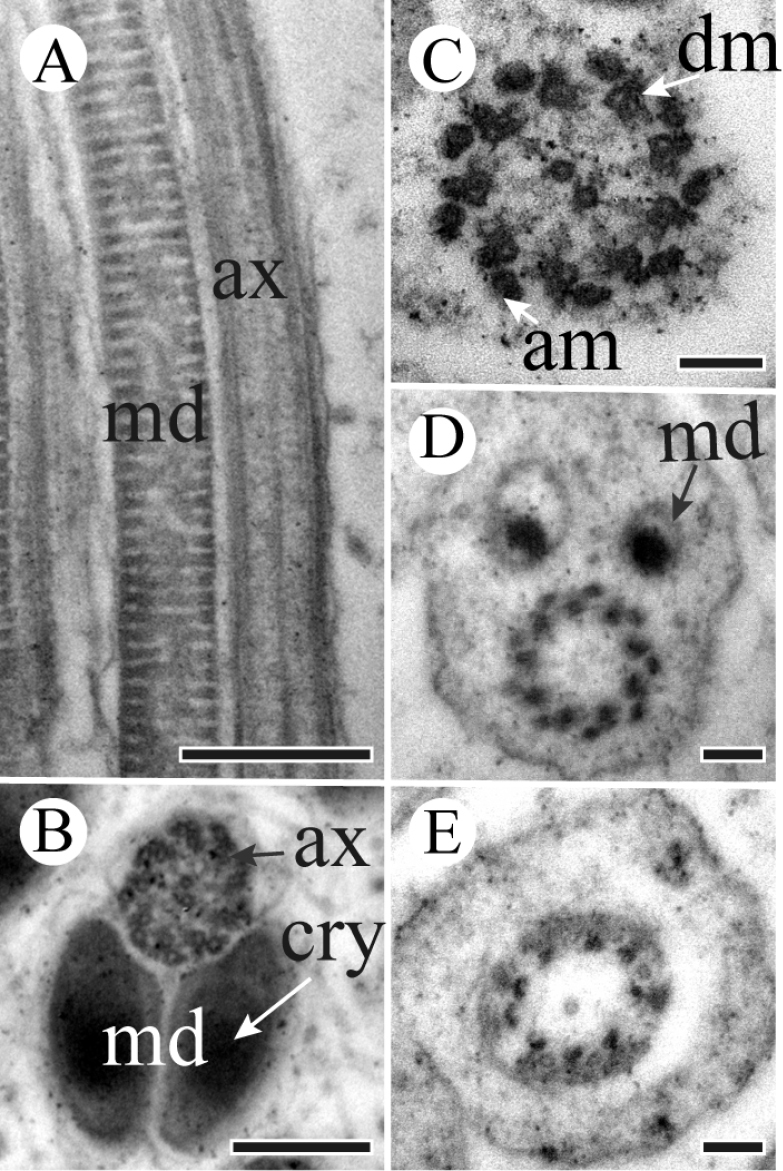
TEM micrographs of *S. yangi* sperm tail region. **A** Longitudinal section of sperm tail, showing axoneme (ax) and mitochondrial derivative (md) **B** Cross-section through the tail region, showing axoneme (ax) and mitochondrial derivatives (md) with crystalline region (cry) **C** Magnified cross-section of axoneme (ax), showing axoneme with a normal 9 + 9 + 2 arrangement of microtubules, i.e., 9 accessory microtubules (am), 9 double microtubules (dm) and 2 central microtubules **D** Cross-section of the terminal end of the sperm tail, showing paired mitochondrial derivatives (md) and axoneme (ax) with 9 accessory microtubules and 9 double microtubules left **E** Cross-section of the terminal end of the sperm tail, showing parts of microtubules of axoneme left. Scale bars: 500 nm (**A**), 200 nm (**B**), 100 nm (**C–E**).

A centriole runs from the flat base of the nucleus, connecting the nucleus and the axoneme (Figure 3A). The flagellum is formed by a 9 + 9 + 2 axoneme (i.e., nine accessory microtubules, nine double microtubules, and two central microtubules) flanked by a pair of equal mitochondrial derivatives with crystalline regions (Figure 4B, C). The derivatives are composed of cristae arranged in an orderly array in longitudinal section (Figure 4A). At the end of the tail, axonemal microtubules appear less well organized and disappeared gradually (Figure 4D, E).

*Karenia caelatata* Distant, 1890

**Figure 5.**
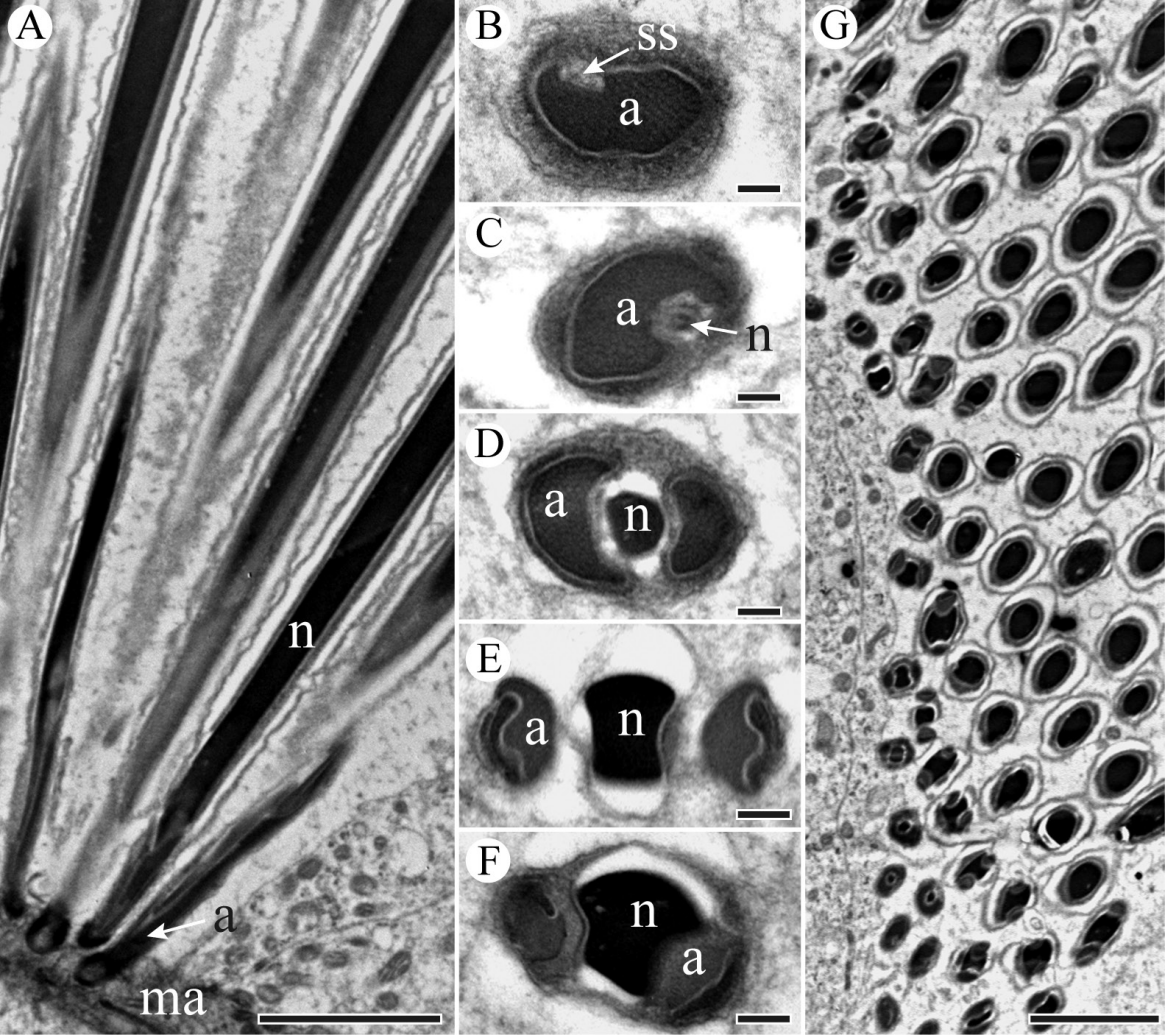
TEM micrographs of sperm head region of *K. caelatata*. **A** Longitudinal section of head region, showing the head region (including acrosome (a) and nucleus (n)) inserted into a homogenous matrix (ma) **B** Cross-section through the acrosome (a), showing the subacrosomal space (ss) located at an eccentric position of acrosome **C** Cross-section through the acrosome, showing the nucleus (n) located at an eccentric position of acrosome **D–F** Cross-sections through the posterior region of the acrosome (a), showing two acrosomal processes and the nucleus (n) **G** Lower magnification of cross-section through spermatodesmata, showing different transverse sections of spermatozoa. Scale bars: 2 μm (**A, G**), 100 nm (**B–F**).

**Figure 6.**
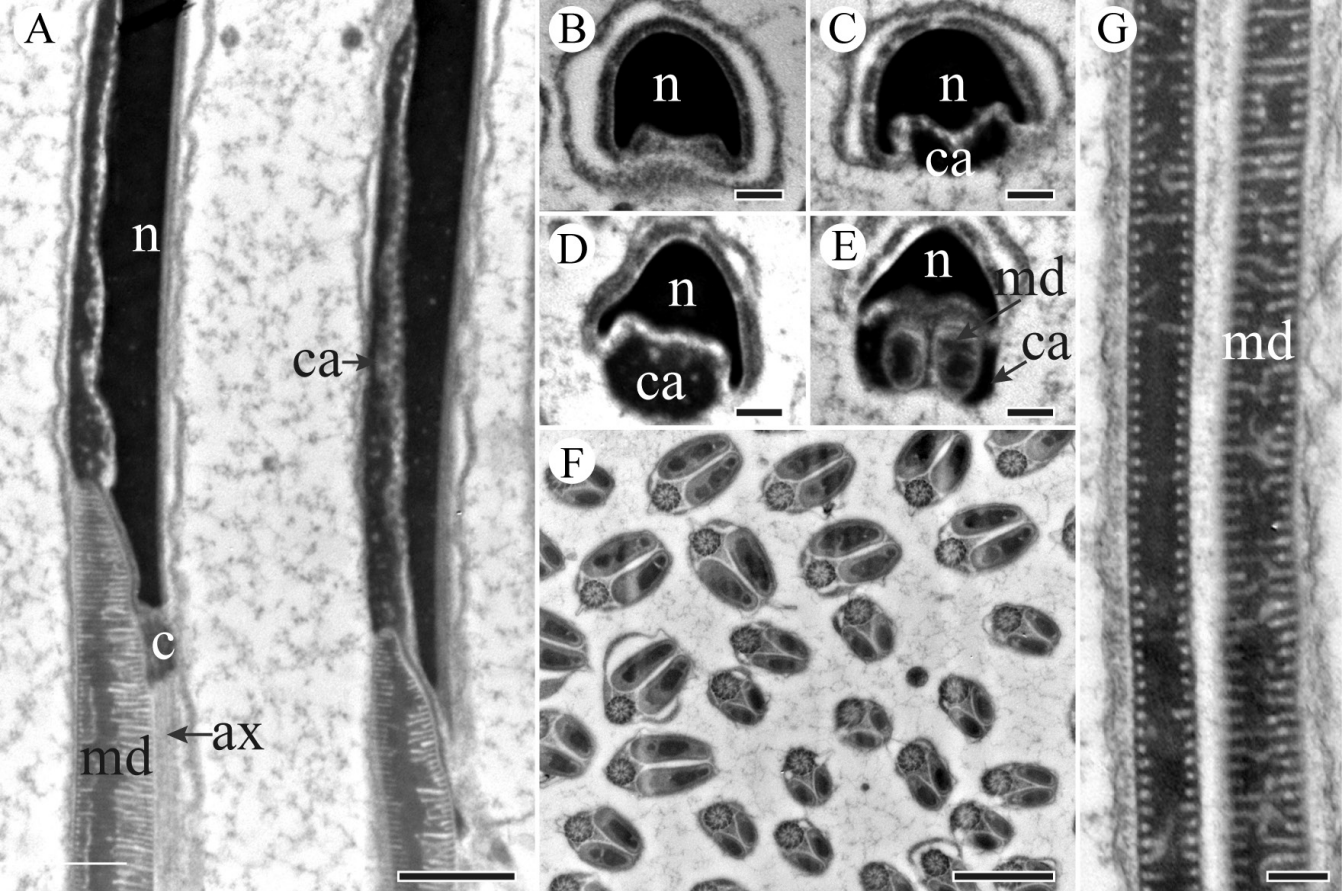
TEM sections through the neck and tail regions of the spermatozoa of *K. caelatata*. **A** Longitudinal section through the neck region showing nucleus (n), centriolar adjunct (ca), centriole (c), axoneme (ax) and mitochondrial derivatives (md) **B** Cross-section through the mid-neck region, showing one side of the nucleus forms two ridges. **C** and **D** Cross-sections through the posterior part of nucleus, showing centriolar adjunct (ca) flanked nucleus (n) **E** Cross-section of the base of the nucleus, showing triangular nucleus (n) and two mitochondrial derivatives (md) embedded into the material of the centriolar adjuncts (ca) **F** Cross-section through sperm tails, showing mitochondrial derivatives with distinct diameters **G** Longitudinal section of sperm tail, showing paired mitochondrial derivatives (md). Scale bars: 1 μm (**A, F**), 200 nm (**B–E, G**).

Spermatozoa are all gathered together, with their conical acrosomes and part of the electron-dense nuclei inserted into a homogenous matrix forming a spermatodesm (Figure 5A). In cross-section, the acrosome is conical, and a sub-acrosomal invagination is eccentric in position anteriorly (Figure 5B). The acrosome forms two processes posteriorly, which flank the anterior part of nucleus (Figure 5C–F). The acrosomal contents are not homogenous in appearance, which are filled with numerous tubular substructures as shown in cross sections (Figure 5B–F). The diameters of the two tubular acrosomal processes increase towards the base of the acrosome, and the nucleus becomes mushroom-shaped in cross sections (Figure 5B–G). The electron-dense centriolar adjunct forms a sheath shape and runs parallel to the posterior part of nucleus (Figure 6A). The posterior segment of the nucleus develops a lateral invagination; the centriolar adjunct is confined within the invagination in cross sections (Figure 6B–E).

The centriole is attached to the base of the nucleus (Figure 6A).The flagellum is composed of an axoneme with a typical 9 + 9 + 2 microtubular pattern and a pair of mitochondria derivatives (Figure 6F, G). The mitochondrial derivates are composed of numerous cristae, which can be seen from the longitudinal section of the tail (Figure 6G). All of derivates with different diameters have a crystalline region in cross-section of tail (Figure 6F).

*Platypleura kaempferi* (Fabricius, 1794)

**Figure 7.**
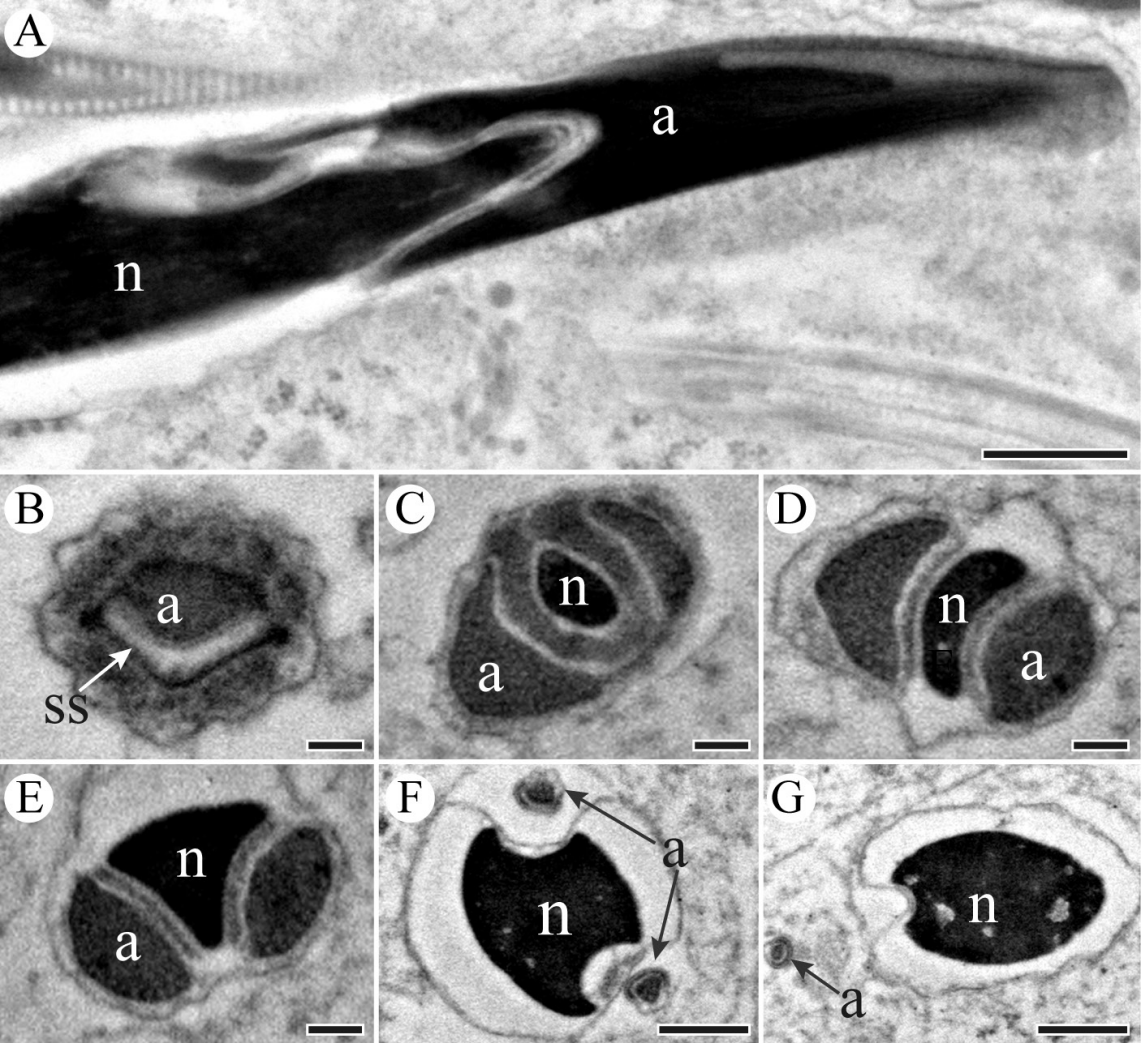
TEM micrographs of sperm head region of *P. kaempferi*. **A** Longitudinal section of sperm head, showing apex of acrosome (a), tapered nucleus (n) **B** Cross-section of the sperm head, showing acrosome (a) and subacrosomal space (ss) **C–F** Cross-sections of the sperm head, showing acrosome (a) and two acrosomal processes with numerous microtubules **G** Cross-section of the sperm head, showing nucleus (n) and an acrosomal process. Scale bars: 500 nm (**A**), 200 nm (**B–G**).

**Figure 8.**
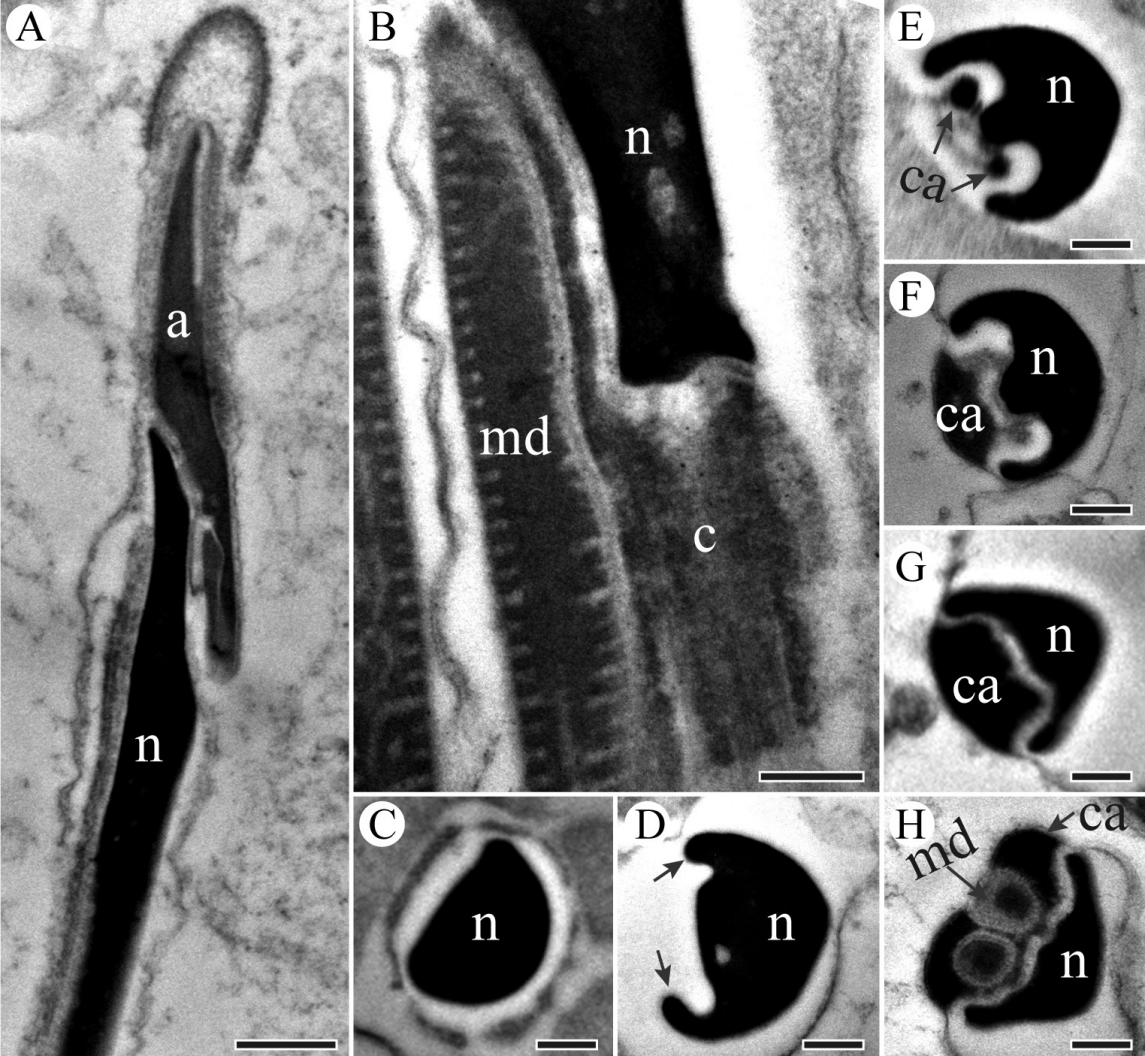
TEM micrographs of sperm neck region of *P. kaempferi*. **A** Longitudinal section showing head region, showing conical acrosome (a) and tapered nucleus (n) **B** Longitudinal section of nucleus-ﬂagellum transition region, showing nucleus (n), mitochondrial derivative (md) and centriole (c) **C** Cross-section of nucleus (n) with a deltoid appearance **D** Cross-section through the mid-neck region, showing an invagination at one side of the nucleus (n) developing two ridges (arrowed) **E–G** Cross-sections through neck region, showing centriolar adjunct (ca) and nucleus (n). **H** Cross-section through the mid-neck region, showing nucleus (n), mitochondrial derivatives (md) and centriolar adjunct (ca). Scale bars: 500 nm (**A**), 200 nm (**B–H**).

Spermatozoa aggregate together with their heads inserted into a homogenous matrix to form a spermatodesm. The head region consists of an acrosome and a compact and homogeneous nucleus (Figs 7A and 8A). The acrosomal contents have tubular substructures (Figure 7B–G). The acrosome is laterally flattened, and an electron-lucent space (viz., subacrosomal space) lies in an eccentric position anteriorly (Figure 7A). The acrosome gradually widens posteriorly, and forms two processes that flank the anterior part of the nucleus in cross-sections (Figure 7C–G). The centriolar adjunct lies beneath the posterior of the nucleus and is parallel to it. The diameter and shape of the centriolar adjunct vary, adapting to the lateral invagination of the nucleus (Figure 8E–G). With the extension of the nucleus, the diameter of nucleus gradually decreases (Figure 8C–H). The centriolar adjunct gradually vanishes where the mitochondrial derivatives emerge (Figure 8H).

**Figure 9.**
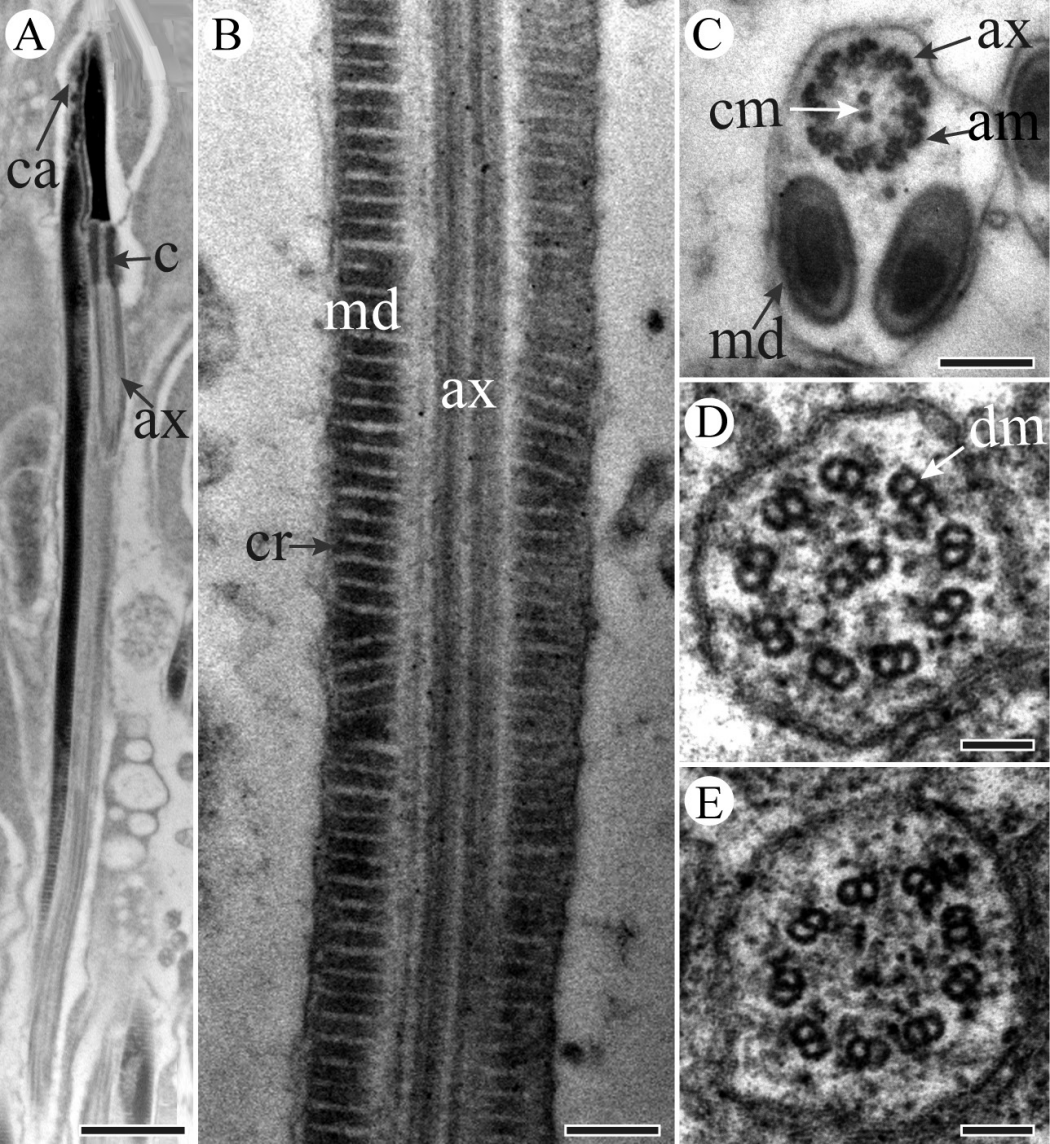
TEM micrographs of sperm tail region of *P. kaempferi*. **A** Longitudinal section through the neck and tail regions, showing nucleus (n), centriolar adjunct (ca), mitochondrial derivative (md), centriole (c) and axoneme (ax) **B** Higher magnification of longitudinal section of sperm tail, showing axoneme (ax) and mitochondrial derivatives with cristae (cr) **C** Cross-section of tail region, showing two mitochondrial derivatives (md) and a 9 + 9 + 2 microtubular pattern (i.e., 9 accessory microtubules (am), 9 double microtubules (dm), and two central microtubules (cm)) axoneme (ax) **D** Higher magnification of cross-section of axoneme (ax), showing 9 double microtubules (dm), and two central microtubules left **E** Higher magnification of cross-section of axoneme (ax) showing only 9 double microtubules (dm) remained. Scale bars: 1 μm (**A**), 200 nm (**B, C**), 100 nm (**D, E**).

The centriole emerges from the base of the nucleus, connecting the nucleus and the axoneme (Figure 8B). In the tail region, the paired mitochondrial derivatives are formed by cristae at the periphery, and a typical axonemal arrangement of 9 + 9 + 2 microtubules is present (Figure 9B and C). Each derivative is positioned laterally to the axoneme and contains an elliptical crystalline region (Figure 9C). Cross-sections through the end of the tail show a progressive loss of microtubules: the 9 accessory microtubules disappear first (Figure 9D), followed by the two central microtubules (Figure 9E). The axoneme extends to almost the end of the sperm tail (Figure 9A).

## Discussion

In this study, a number of similarities are revealed in the mature spermatozoa of *S. yangi*, *K. caelatata* and *P. kaempferi*. The motile spermatozoa, all aggregated into bundles, intrude into a homogenous matrix to form a spermatodesm. The spermatozoa of each species can be divided into two or three types based on their total length, nucleus length, and tail lengths (viz., polymegaly). There is a conical acrosome with a subacrosomal space in an eccentric position, and the acrosome sits above the anterior part of the nucleus. The centriolar adjunct is located at the postero-lateral invagination of the nucleus and is parallel to it. In the tail region, two equal mitochondrial derivatives with electron-dense crystalline regions comprise cristae, which are arranged in an orderly array. A single axoneme displays a 9 + 9 + 2 microtubule arrangement. The mitochondrial derivatives and the axoneme both extend to almost the end of the tail. There is no accessory body in the sperm tail. These features are all likely common to spermatozoa of other investigated cicadas (e.g., Folliot and Maillet 1970, Kubo-Irie et al. 2003, Chawanji et al. 2005, 2006).

Although the sperm ultrastructures of these three cicada species have some similarities, the centriolar adjunct of *S. yangi* presents a different appearance, i.e., with a granular substructure. In many insects, the centriolar adjunct has been identified as derived from additional pericentriolar material (PCM) deposited beneath the nucleus at the end of spermiogenesis (Dallai et al. 2016a). The centriolar adjunct is apparently an apomorphy of Insecta (Dallai et al. 2016b). In Dicondylia of the Insecta the centriolar adjunct and the accessory bodies may be variably developed, and their size and shape are important characteristics for taxonomy (Dallai et al. 2016a). Cicadas are classified into two families, Cicadidae and Tettigarctidae (Moulds 2005, Marshall et al. 2018), with the former being divided into four subfamilies (Cicadinae, Cicadettinae, Tibicininae, and Tettigomyiinae) (Marshall et al. 2018). So far, the centriolar adjunct of spermatozoa in Cicadinae has been found to be composed of homogenous, moderately electron-dense material (Folliot and Maillet 1970, Kubo-Irie et al. 2003, Chawanji et al. 2005). In our present study, the structure of the centriolar adjunct in spermatozoa of *P. kaempferi* and *K. caelatata* is consistent with previous descriptions for the Cicadinae. In the Cicadettinae and Tettigomyiinae, the centriolar adjunct of the spermatozoa is lamellar (Chawanji et al. 2006). In our study, we found that the centriolar adjunct of *S. yangi* is granular, which is different from that of other cicada species, indicating that spermatozoa in Tibicininae may have their own characteristics in comparison with other cicadas. Chawanji et al. (2005) described vesicular-like structures associated with the centriolar adjunct in some sections of spermatozoa of the cicada *P. hirtipennis* (Germar, 1834), but the centriolar adjunct itself is not granular, which is different to the granular centriolar adjunct of *S. yangi*. This characteristic may add more information for spermatology of Tibicininae and, together with other results, may inform future studies on the phylogeny of Cicadoidea.

Cicadas of the genus *Karenia*, remarkably without timbals, are currently placed in the Cicadinae. This is the only genus of the tribe Sinosenini. The systematic placement of this tribe remains controversial (Boulard 1973, 1988, 2008, 2001, 2013, Chou et al. 1997, Moulds 2005, Wei et al. 2009, Marshall et al. 2018). Species of this group are restricted to southwestern China, Myanmar and Vietnam (Wei et al. 2009, Pham and Yang 2012). Moulds (2005) attributed *Karenia* to the subfamily Cicadettinae, but this genus and other probably related taxa were not included in his morphological phylogenetic analyses. Boulard (2008), Wei et al. (2009) and Pham and Yang (2012) followed Moulds (2005), attributing this genus into the Cicadettinae. However, Boulard (2013) and Marshall et al. (2018) put this genus in the Cicadinae. Morphologically, the metanotum of *Karenia* is distinctly concealed by the cruciform elevation at the dorsal midline, which is the same as species in Cicadinae, but is different from members of Cicadettinae whose metanotum is partly visible at dorsal midline (Moulds 2005, 2012). Furthermore, in Cicadettinae the uncus is duck-bill shaped and undeveloped, and the pair of claspers are well developed; while in Cicadinae the uncus is well developed with uncal lobes much swollen and elongated, and the claspers are usually degenerate or even disappeared (Moulds 2005, 2012). The strongly swollen uncus in *Karenia* is similar to that in the Cicadinae. In some species of Cicadettinae, the centriolar adjunct presents as a lamellate substructure (Chawanji et al. 2006). However, in our study, there is no such substructure in the centriolar adjunct of the spermatozoa of *K. caelatata*. Coupled with the above-mentioned morphological characters and the morphology of ovipositors (Zhong et al. 2017), antennae (Wang et al. 2018, in press) and Malpighian tubules (Li et al. 2015), our results confirm that it is reasonable to place this genus in Cicadinae, which is consistent with the results of Marshall et al. (2018) based on molecular data.

Spermatozoa possess more than one size type (viz., polymegaly), which has been described widely within the Insecta. For example, some species of vinegar flies (Diptera, Drosophilidae) and stalk-eyed flies (Diptera, Diopsidae) produce two discrete lengths of nucleated sperms (Snook et al. 1994, Pasini et al. 1996, Presgraves et al. 1999). In the Cicadomorpha, all of the 13 previously investigated species of Cicadoidea can produce more than one size type of spermatozoa reflected in nuclear length and total length within and between species (Kubo-Irie et al. 2003, Chawanji et al. 2005, 2006). In addition, a distinct correlation between nuclear length and total length was found in the spermatozoa of cicada *G. nigrofuscata* (Kubo-Irie et al. 2003). However, Chawanji et al. (2005, 2006) found the nucleus length and total length of spermatozoa have no significant correlations in their examined species. In our study, the three examined species also produce two or three distinct size types of spermatozoa, and there is a weak (in *S. yangi*) or modest (in *K. caelatata* and *P. kaempferi*) correlation between the nuclear length and tail length within a species (Table 3). In contrast, polymegaly of spermatozoa does not appear in the Cicadellidae and Cercopoidea within Cicadomorpha. The production of only one size type of spermatozoa has been revealed in investigated species of Membracoidea (Cruz-Landim and Kitajima 1972, Araújo et al. 2010, Zhang and Dai 2012, Su et al. 2014). Although the sperm length varies within individual males of *Locris transversa* (Cercopidae), this variation has no statistical significance, which was also observed in other two cercopids (Folliot and Maillet 1970). Hodgson et al. (2016) presumed that polymegaly may be an apomorphy of Cicadoidea within Cicadomorpha based on the study of *Locris transversa* (Cercopidae). Therefore, our results, coupled with other previously related studies, suggest that polymegaly is a narrow occurrence in the Cicadomorpha.

The results of our study provide more clues for further studies of classification and phylogeny of the Cicadoidea. There may also be some ultrastructural features that can be used as morphological evidence for the phylogeny of the Cicadomorpha.
